# Ultrastructural study of adult *Haemonchus contortus* exposed to polyphenol-rich materials under *in vivo* conditions in goats

**DOI:** 10.1051/parasite/2019065

**Published:** 2019-11-18

**Authors:** Cintli Martínez-Ortiz-de-Montellano, Juan Felipe de Jesús Torres-Acosta, Isabelle Fourquaux, Carlos Alfredo Sandoval-Castro, Hervé Hoste

**Affiliations:** 1 Departamento de Parasitología, Facultad de Medicina Veterinaria y Zootecnia, Universidad Nacional Autónoma de México Colonia UNAM, CU, Delegación Coyoacán CP 04510 Ciudad de México México; 2 Facultad de Medicina Veterinaria y Zootecnia, CCBA, Universidad Autónoma de Yucatán Km. 15.5 carretera Mérida-Xmatkuil 97100 Mérida Yucatán México; 3 CMEAB, Faculté de Médecine de Rangueil, Université Paul Sabatier 31062 Toulouse Cedex France; 4 INRA UMR 1225 IHAP INRA/ENVT 23 chemin des Capelles 31076 Toulouse Cedex France; 5 Université de Toulouse, ENVT, UMR 1225 31076 Toulouse Cedex France

**Keywords:** Ultrastructural changes, *Haemonchus contortus*, Polyphenol-rich plants, Goats

## Abstract

This study assessed the ultrastructural changes caused in adult *Haemonchus contortus* obtained from goats fed fodder based on polyphenol-rich plants *Lysiloma latisiliquum* or *Onobrychis viciifolia* or from goats drenched with quebracho extract, *Schinopsis* spp. The *H. contortus* were obtained from artificially infected goats used as models to investigate the anthelmintic effect of feeding or drenching with the polyphenol-rich materials. Nematode populations were exposed to polyphenol-rich plant materials by feeding host goats for 8 consecutive days (D28 to D35 post*-*infection) with (a) *L. latisiliquum* fodder at 800 g fresh basis/day, (b) *O. viciifolia* fodder offered *ad libitum*, and (c) drenched with a solution containing quebracho extract (90 g/day). Meanwhile, control *H. contortus* were obtained from goats fed polyphenol-free diets. The *H. contortus* specimens were recovered from the goats on D36 post-infection, and transmission electron microscopy (TEM) was used to identify ultrastructural changes. *In vivo* exposure to different polyphenol-rich plant materials caused vacuolization of the nematodes’ intestinal, muscular and hypodermal cells. These alterations represent the first evidence of cell damage caused in *H. contortus* when hosts were fed or drenched with polyphenol-rich materials. Ultrastructural changes affecting several types of cells could explain modifications in worm motility and nutrition, eventually affecting *H. contortus* reproductive success. This study contributes to our understanding of the mechanisms of action of polyphenol-rich plants against *H. contortus* when given as nutraceuticals to goats.

## Abstract (fr)

Cette étude a examiné les lésions ultrastructurales provoquées chez les adultes d’*Haemonchus contortus* obtenus chez des chèvres alimentées par des fourrages riches en polyphénols, *Lysiloma latisiliquum* ou *Onobrychis viciifolia*, ou des chèvres recevant des extraits de quebracho, *Schinopsis* spp. Les *H. contortus* ont été obtenus à partir de chèvres artificiellement infestées pour vérifier les effets anthelminthiques de l’alimentation ou l’administration de ressources riches en polyphénols. Les populations de nématodes ont été exposées à des ressources végétales contenant des polyphénols chez des hôtes infestés recevant pendant 8 jours (J28 à J35 après infestation) : (a) un fourrage à base de *L. latisiliquum* (800 g/jour), (b) un fourrage à base de sainfoin (*O. viciifolia*) offert *ad libitum,* et (c) une solution d’extrait de quebracho (90 g/jour). Pendant ce temps, une population témoin d’*H. contortus* a été obtenue à partir de chèvres alimentées avec un régime à base de fourrages sans polyphénols. Des échantillons d’*H. contortus* ont été obtenus à partir des divers groupes de chèvres à J36 après infestation, et la microscopie électronique à transmission a été utilisée pour identifier les modifications ultrastructurales. L’exposition *in vivo* à différents matériaux végétaux riches en polyphénols chez l’hôte a provoqué chez les nématodes une vacuolisation des cellules intestinales, musculaires et hypodermiques. Ces altérations représentent la première preuve de lésions cellulaire provoquées chez *H. contortus* lorsque l’hôte reçoit des ressources riches en polyphénols. Les changements ultrastructuraux affectant divers types de cellules pourraient expliquer des modifications fonctionnelles de motilité et de nutrition des vers, et finalement modifier le succès reproducteur d’*H. contortus*. Cette étude contribue à la compréhension des mécanismes d’action des plantes contenant des polyphénols utilisées comme alicament contre *H. contortus*.

## Introduction

In addition to their nutritional value, plants and their bioactive compounds have also been used to treat various ailments in humans or animals, because of their medicinal properties. Recently, the anthelmintic (AH) activity of polyphenol-rich (PR) plants has been explored as a possible novel tool for the sustainable control of gastrointestinal nematodes (GINs) of ruminants in this era of resistance to conventional drugs. The biological effects of PR fodder against adult *Haemonchus contortus* populations were first reported for quebracho extract [28] and were confirmed for PR legume plants from temperate regions: e.g., *Onobrychis viciifolia* [3, 15, 16, 25, 29, 30] or *Lespedeza cuneata* [20, 23, 33, 34]. Similar results were obtained for tropical legume trees such as *Lysiloma latisiliquum* [9, 26] and *Havardia albicans* [14]. The most frequent effect reported for animals fed with these plant materials is the reduction of parasite faecal egg excretion, which has been related either to a reduction in worm numbers [15, 16] or reduced individual worm fertility [14–16, 25, 26, 29, 30].

Based on the hypothesis of the direct pharmacological-like effects of the secondary plant metabolites against GIN [18, 21], some studies aimed at better understanding the mode of action of PR materials, which could explain the changes in function and structure of third-stage larvae [7–10]. In contrast, the mechanisms explaining the anthelmintic effects against adult worms, resulting from the consumption of plant materials containing bioactive compounds, remain largely unknown. Furthermore, the target organs within *H. contortus* that may become affected when ruminants eat PR materials are still unknown. Previous work, using scanning electron microscopy (SEM), showed alterations in the external structure of the adult *H. contortus* cuticle as well as material aggregated around the buccal capsule when exposed to PR plant materials under *in vitro* and *in vivo* conditions [23, 27]. There is no evidence on the effect on the ultrastructure of adult GINs exposed to PR materials consumed by ruminants. A recent study with *Acacia oxyphylla* bark extract revealed ultrastructural changes in the tegument interface of the cestode *Raillietina echinobothrida*; however, these changes were not associated with any specific compound [13]. The present trial aimed at assessing and comparing the ultrastructural changes caused in adult *Haemonchus contortus* obtained from goats fed fodder of PR plants (the tropical *Lysiloma latisiliquum* or the temperate *Onobrychis viciifolia*) or goats drenched with quebracho extract (*Schinopsis* spp.), in order to better understand the mode of action of PR resources.

## Materials and methods

The present study was designed to obtain adult *H. contortus* from donor goats fed/drenched with PR materials. The study also included goats that were used as donor hosts for parasites fed control polyphenol-free diets.

The study was performed in two locations. The first part of the protocol was undertaken at the Facultad de Medicina Veterinaria y Zootecnia, Universidad Autónoma de Yucatán (FMVZ-UADY), Mérida, México, and the second was performed at the Unité Mixte de Recherche 1225 Interactions Hôtes Agents Pathogène, Institut National de la Recherche Agronomique – École Nationale Vétérinaire de Toulouse, France (UMR 1225 IHAP, INRA-ENVT).

### Artificial infection of experimental goats with *Haemonchus contortus*

In the first location of the study, four experimental goats were artificially infected with 3000 L_3_ of a Mexican strain of *H. contortus* obtained from Centro Nacional de Investigación Disciplinaria de Parasitología Veterinaria, Instituto Nacional de Investigación Forestal, Agrícola y Pecuaria (CENID-PAVET-INIFAP). Six goats used in the second location of the study were artificially infected with 3000 L_3_ of a French caprine strain of *H. contortus* INRA-ENVT*.*

### Exposure of donor goats to PR diets

In the protocol performed in Mexico, the parasites were exposed to fresh leaves of *L. latisiliquum* (tzalam) (Fabacae) harvested during the rainy season from the tropical forest in Merida, Yucatan, Mexico. Two donor animals were offered 800 g of fresh tzalam leaves from day 28 post infection (PI) and received the foliage for eight consecutive days. The plant species was selected firstly because goats readily consume this fodder, and also for the anthelmintic activity of its secondary compounds recorded against *H. contortus* under *in vitro* [1, 35] and *in vivo* conditions [9, 26]. During the same period post-infection (D28 to D35) the control *H. contortus* were maintained within two experimental goats receiving a polyphenol-free diet. The ethics committee of the FMVZ-UADY approved the experimental protocol with animals (agreement CB-CCBA-D-2014-003).

When the protocol was performed in France, the parasites within four experimentally infected goats were either exposed to sainfoin hay (*O. viciifolia*) consumed *ad libitum* or exposed to an oral suspension containing quebracho extract (90 g/day) for eight consecutive days post-infection (D28 to D35). The *O. viciifolia* fodder was selected because it is known to contain secondary compounds with anthelmintic activity against *H. contortus* [7, 10, 15, 16, 25], and the same is true for the commercially available extract of *Schinopsis* spp. [4, 5, 24]. In this second protocol of the study, there was another control *H. contortus* population within two goats that received a polyphenol-free diet. The facilities hosting the animals and trial conduct met ethical and welfare rules applicable in France (agreement SSA No. 115 of 15 December 2014).

### Recovery of adult *Haemonchus contortus* from donor goats

On day 35 PI, all the goats at both experimental sites were slaughtered according to ethical rules by injection of 3.65 g of a pentobarbital dose intravenously. Then, abomasa were immediately removed in order to collect adult *H. contortus* from each animal. The worms were fixed immediately, as described below.

### Transmission electron microscopy (TEM)

Adult female worms, recovered from each animal representing a model of feeding/drenching were fixed separately in 2% glutaraldehyde-sodium cacodylate buffer (pH 7.4, 0.1 M) for at least 48 h at 4 °C. The worms were preserved at 4 °C until further processing in the “Centre de Microscopie Electronique Appliquée à la Biologie” (CMEAB) of the University Paul Sabatier, Toulouse. The overall length of fixation of the worms with 2% glutaraldehyde-sodium cacodylate buffer at 4 °C was approximately 2 weeks for the French samples, and close to 3 weeks for the Mexican samples. However, for the Mexican samples, the cold chain was interrupted for 36 h during the transport.

In CMEAB, worms were first washed overnight in 0.2 M sodium cacodylate buffer. Then, each *H. contortus* was gently cut up into three parts in order to allow better penetration of the post-fixative, dehydrating solutions and resin. The anatomical part selected for the study was the posterior end of adult female worms because it was thought that this would permit examination of three organs of *H. contortus*, namely the intestine, uterus and ovaries. Worms were then post-fixed for 1 h at room temperature (25 °C) with 1% OsO_4_ in 0.2 M sodium cacodylate buffer.

Samples were dehydrated through a series of graded ethanol solutions (30%, 50%, 70% and 95%, 10 min each; 100%, 3 × 15 min) and were then embedded in London Resin White “LRW” (EMS-Euromedex, France) through successive substitution washes, consisting in dilutions of ethanol in 1/3 LRW (5 h at 4 °C) and 2/3 LRW (overnight at −20 °C to prevent spontaneous polymerisation). This was followed by impregnation in pure LRW (30 h in 3 washes at 4 °C). The polymerization lasted for 48 h at −20 °C under UV light.

Prior to preparation of samples for TEM examination, semi-thin 0.5 μm sections were cut, using Ultracut Reichert Jung microtome, and stained with Methylene Blue Azur II. These sections were observed at 100× magnification with an optical microscope to evaluate changes in different tissues of the worms and to orient the preparation of ultrathin sections. Finally, 70-nm thick sections of worms were cut on Ultracut Reichert Jung microtome and mounted on 100-mesh, collodion-coated copper grids prior to staining with 2% uranyl acetate in aqueous solution (2 min) and Reynold’s lead citrate (7 min). Examinations were carried out on a Hitachi HU12A TEM at an accelerating voltage of 75 kV.

## Results

### Histological results on semi-thin sections

**Figure 1:**
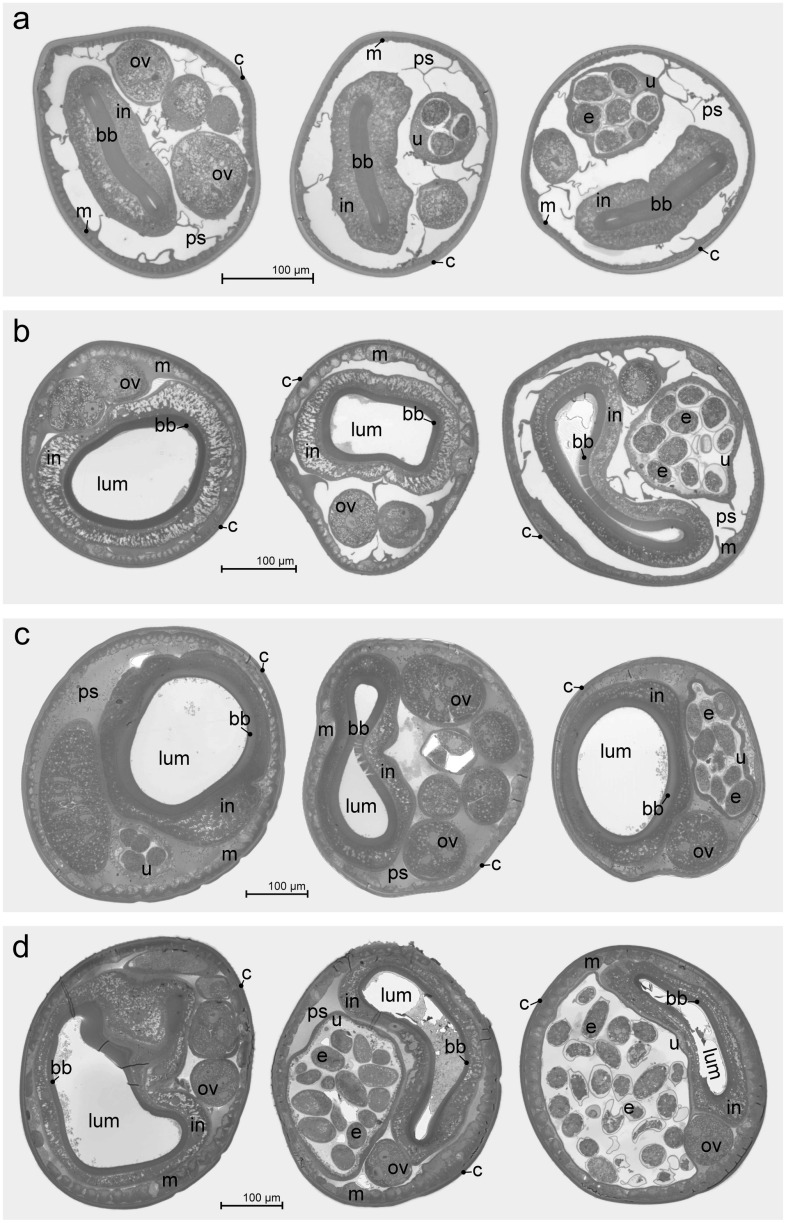
Differences between *Haemonchus contortus* sections obtained either from goats fed a polyphenol-free diet (a), goats fed with tzalam fodder (b), drenched with quebracho (c) or fed sainfoin fodder (d). Cross-section of worms show cuticle (c), muscle (m), intestine (in), lumen of intestine (lum), brush border (bb), ovary (ov), uterus (u), egg sections (e) and pseudocoele (ps). Stained with Methylene blue (Azur II).

The semi-thin sections of the posterior end of *H. contortus* females exposed to the control diet are shown in Figure 1a. The comparison with *H. contortus* exposed to PR plants (tzalam or sainfoin) or quebracho extract (Figs. 1b, 1d and 1c, respectively) showed, at this level of observation, that the intestinal epithelium and the muscle cells of the worms were the two main tissues that appeared consistently affected by the plant materials tested. However, some differences were found depending on each tannin-containing resource. Cell vacuolisation zones were detected in the intestinal epithelium of parasites exposed to the three plants, but the lesions in the worms obtained from goats fed the tzalam or sainfoin foliage appeared more severe and extensive than those generated in parasites from goats drenched with quebracho (Figs. 1b, 1d and 1c, respectively).

### TEM results in nematodes treated with PR materials

#### Cuticle and muscular cells

**Figure 2:**
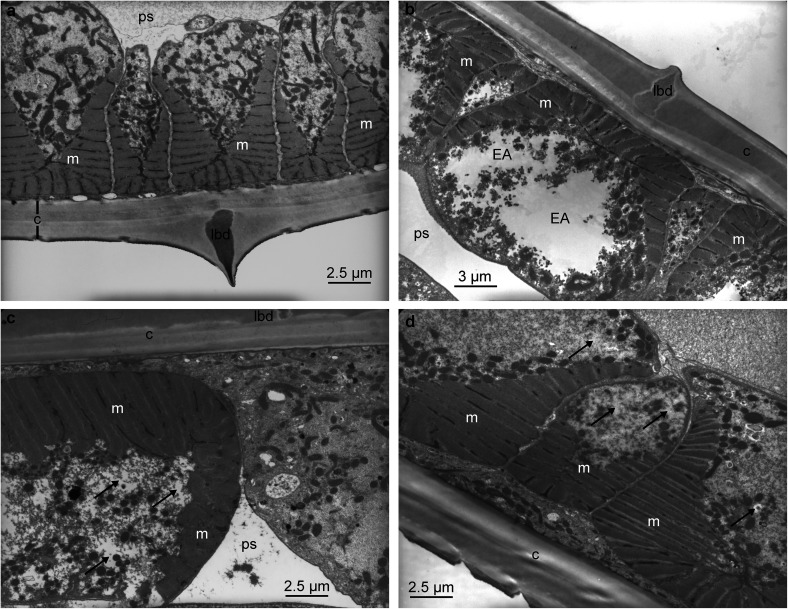
Transmission electron microscopy (TEM) of cuticle and muscle of *Haemonchus contortus* obtained from goats fed (a) control (polyphenol-free diet), (b) tzalam fodder, (c) quebracho drench or (d) sainfoin fodder. The illustrations include the longitudinal body ridge (lbd), cuticle (c), muscle cells (m), pseudocoele (ps). The main cellular lesions are electrolucent areas (EA), indicated by arrows in Figures 2c and 2d.

Figure 2a shows the normal ultrastructure of the cuticle and muscular tissue from control worms (obtained from goats fed a polyphenol-free diet) including normal longitudinal body ridge, cuticle, parts of several muscle cell and pseudocoele. Lesions were not observed in the cuticle of worms exposed to different PR materials (Figs. 2b, 2c and 2d). The muscle cells of *H. contortus* obtained from goats fed PR diets showed vacuolisation, with the main changes being observed in the worms from tzalam fed goats (Fig. 2b). The latter showed the formation of large irregular electrolucent areas (Fig. 2b). Vacuolisation was milder in worms obtained from quebracho-treated goats (Fig. 2c). The *H. contortus* from sainfoin-fed goats showed more electrolucent areas in the cytoplasm, with less vacuolisation (Fig. 2d) compared to control worms.

#### Intestinal cells

**Figure 3:**
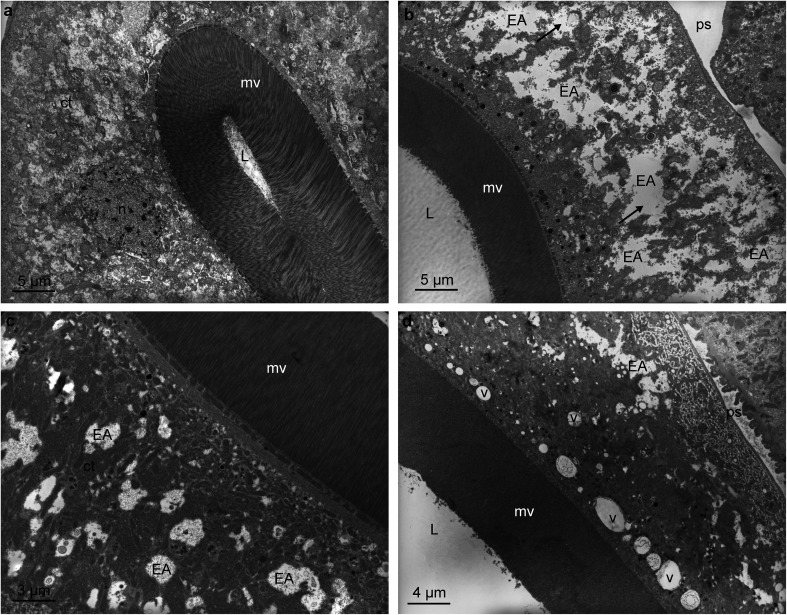
Transmission electron microscopy (TEM) of intestinal cells of *Haemonchus contortus* obtained from goats either (a) on control (polyphenol-free) diet, (b) fed tzalam fodder, (c) drenched with quebracho or (d) fed sainfoin fodder. The illustrations include nucleus (n), cytoplasm (ct), microvilli (mv), lumen (L), pseudocoele (ps) and the presence of vacuoles (v) and electrolucent areas (EA) indicated by arrows in Figure 3b.

The normal ultrastructure of *H. contortus* intestinal cells from the control goat is shown in Figure 3a. The worms exposed to the respective PR fodders showed large intra-cytoplasmic electrolucent areas (Figs. 3b, 3c and 3d). Larger disrupted areas were observed in the worms exposed to tzalam (Fig. 3b) compared to those exposed to quebracho (Fig. 3c) and sainfoin (Fig. 3d).

#### Uterine cells

No observable differences were noted in the uterus and eggs of *H. contortus* from goats fed the control diet and PR fodder, regardless of the feed source. Therefore, no illustrations are presented.

## Discussion

To our knowledge, this is the first study examining ultrastructural changes caused by PR materials on adult *H. contortus* female worms obtained under *in vivo* conditions from goats. The consumption of PR plant materials may play a role in the control of GIN infections [19]. The biological effects of PR fodders against populations of adult *H. contortus* were first reported with quebracho [28] and different tannin-containing legumes from temperate areas such as *O. viciifolia* [3, 8, 25] or *L. cuneata* [20, 23, 33, 34]. Similar effects have been reported for tropical legume trees such as *L. latisiliquum* [26] and *H. albicans* [14]. These studies showed that animals consuming PR materials had reduced parasite egg excretion, which has been related either to a reduction in the worm numbers [15, 16], and/or reduced individual worm fertility [14–16, 23, 25, 26, 29, 30].

The effects of PR plants on the external structure of *H. contortus* have been studied previously by SEM [23, 26]. Their study provided two major findings: (a) alteration of the cuticle (loosening of its smooth surface) and (b) the presence of aggregates around the buccal capsule. It has been hypothesized that structural changes might disrupt the process of worm nutrition, resulting in stunted growth and reduced fecundity when compared to control worms [14, 26].

The current study on the ultrastructure of *H. contortus* aims at complementing previous SEM observations on worms exposed to different PR materials. As with earlier SEM observations on *H. contortus*, the different plant materials, probably containing different types of secondary plant compounds, including a variety of polyphenolic compounds such as condensed tannins, seem to provoke similar ultrastructural changes but with different magnitudes. The use of tzalam seemed to cause more marked and extensive changes in *H. contortus* female populations than sainfoin fodder or quebracho extracts. The main consequences concerned both the muscle and intestinal cells and involved extensive vacuolisation of the cytoplasm. Changes on these two tissues were observed even on semi-thin sections (Figs. 1b, 1c and 1d). Sainfoin activity results mainly from prodelphinidin-rich tannins [31] and the effect of quebracho may be due to the profisetinidin content [22]. Quercitrin and arbutin contained in tzalam could be assigned as two of the main metabolites related to the AH activity against *H. contortus* L_3_ larvae [17].

The cytoplasmic vacuolisations described can be interpreted as signs of disturbances in cellular functions, possibly due to imbalance of fluid exchanges between the intestinal and pseudocoelomic space (Figs. 1b, 1c, 1d and 3), or between the muscle and the pseudocoelomic space. Similar changes were reported in the cestode *Raillietina echinobothrida* when exposed to a tannin rich material [13].

The ultrastructural changes found in the intestinal cells might be due to the ingestion of bioactive compounds (including tannins and other polyphenols) by the worms, and the resulting direct contact between the bioactive compounds and the intestinal cells. Meanwhile, we hypothesize that these changes found in the muscle cells might result from the contact and/or passage (active or passive) of plant secondary compounds through the worm′s cuticle or the blockade of cell metabolism processes possibly due to changes in cuticle permeability.

Finally, many changes found in the present study were similar to those reported with conventional synthetic AH drugs. The vacuolisation of various organelles and cytoplasm has been recorded in *H. contortus* treated with closantel and these lesions were also related to fluid imbalance [32]. Autophagic processes in intestinal cells have also been described in different nematodes treated with benzimidazoles [6, 36]. Moreover, some specific damage to the different organs, for example brush border disruption in intestinal cells or muscle degradation, has also been described using conventional AH treatments [2, 32].

The present results may provide a useful starting point to further study the direct effect of non-conventional, natural AH compounds, especially PR nutraceuticals. More studies are necessary to better understand the role of bioactive secondary plant compounds involved in the ultrastructural changes reported here. The nature of tannins [31] and a series of flavonoids [7] should be considered with the same methods to explore the possible consequences on the two different cell types that were clearly affected in the *H. contortus*. Other secondary plant compounds should also be investigated besides polyphenols. For example, recent studies have suggested that tannins might not be the sole compounds involved in the AH effect of *L. latisiliquum* extracts [17] or that interactions between different polyphenolic compounds can occur [22].

It is also important to confirm whether the ultrastructural changes found in the nematodes would be reversible once the PR material is withdrawn from the host’s diet. Similarly, it will be essential to differentiate between cell death, apoptosis or necrosis in the worm cells. In the light of some recent evidence showing differential sensitivity to PR extracts in *H. contortus* from different geographic origins [11, 12], TEM would be useful to study differences in lesions between different parasite isolates.

In conclusion, the consumption of the two PR fodders (*L. latisiliquum* or *O. viciifolia*) or the oral administration of quebracho extract (*Schinopsis spp*) in goats caused ultrastructural changes in two main cell types: the intestinal and muscular cells of *H. contortus.* These changes appear to be the main target of the bioactive secondary plant compounds contained in the three different PR plant materials tested *in vivo*. Ultrastructural changes in muscular and intestinal cells could explain changes in worm motility and nutrition, possibly affecting the reproductive success of *H. contortus*. These results contribute to our understanding of the direct AH mechanisms of action against *H. contortus* when animals are fed bioactive PR materials.
